# An Outbreak of Multidrug-Resistant *Shigella flexneri* Serotype 2a Among People Experiencing Homelessness in Vancouver

**DOI:** 10.3390/tropicalmed10050120

**Published:** 2025-04-28

**Authors:** Victor Leung, Gordon Ritchie, Aleksandra Stefanovic, Colin Lee, Sam Chorlton, Nancy Matic, Marc G. Romney, Althea Hayden, Christopher F. Lowe

**Affiliations:** 1 Division of Medical Microbiology, Department of Pathology and Laboratory Medicine, Providence Health Care, Vancouver, BC V6Z 1Y6, Canada; gritchie@providencehealth.bc.ca (G.R.); astefanovic@providencehealth.bc.ca (A.S.); nmatic@providencehealth.bc.ca (N.M.); mromney@providencehealth.bc.ca (M.G.R.); christopher.lowe@ubc.ca (C.F.L.); 2 Division of Infectious Diseases, Department of Medicine, Providence Health Care, Vancouver, BC V6Z 1Y6, Canada; 3 Department of Pathology and Laboratory Medicine, University of British Columbia, Vancouver, BC V5Z 1M9, Canada; sam@bugseq.com; 4 Lower Mainland Pharmacy Services, Providence Health Care, Vancouver, BC V5Z 3N9, Canada; clee88@providencehealth.bc.ca; 5 Public Health, Vancouver Coastal Health, Vancouver, BC V5S 1M9, Canada; althea.hayden@vch.ca

**Keywords:** *Shigella* spp., shigellosis, outbreak, homeless

## Abstract

**Background**: We describe a community-based outbreak of multidrug-resistant *Shigella flexneri* serotype 2a among people experiencing homelessness (PEH) in Vancouver’s Downtown Eastside during the COVID-19 pandemic. **Methods**: In this observational cohort study, we followed the Outbreak Reports and Intervention Studies of Nosocomial Infection (ORION) reporting guidelines. We identified cases by laboratory surveillance and collected demographic and clinical data from the medical charts or patient interviews. We implemented enhanced surveillance and disseminated testing and management guidelines. *Shigella flexneri* isolates were serotyped, and whole-genome sequencing was performed. **Results**: We identified 101 confirmed cases of *Shigella flexneri* 2a (80% male; median age 43) between 31 January and 16 December 2021. All the affected individuals experienced homelessness, and substance use disorder was the most common comorbidity (88%). Five patients required ICU hospitalization, and one death occurred within 30 days. Core-genome multilocus sequence typing analysis confirmed a clonal outbreak. All *S. flexneri* isolates were phenotypically and genotypically multidrug-resistant. **Conclusions**: COVID-19 exacerbated longstanding public health concerns around the dearth of hygiene and sanitation resources available to PEH. Preventing similar outbreaks will require addressing these risks and finding solutions to the crisis of homelessness in Canada.

## 1. Introduction

*Shigella* spp., the cause of bacillary dysentery or shigellosis, is the second leading cause of diarrheal mortality among all ages globally [1]. Shigellosis typically presents with mild to moderate gastrointestinal symptoms; however, severe cases can progress to secondary bacteremia and colonic rupture. Treatment options are becoming increasingly limited with the rising incidence of multidrug-resistant (MDR) and extensively drug-resistant (XDR) strains [2,3].

### 1.1. Setting

The Vancouver Downtown Eastside (DTES) is home to the city’s marginalized populations including people with mental illness, substance use disorders, and people experiencing homelessness (PEH) or unstable housing in single-room-occupancy (SRO) hotels [4]. Since 2018, there has been more widespread displacement, ultimately leading to the highest homelessness rates in the city [5]. During the COVID-19 pandemic, access to washrooms in cafes, restaurants, and city-run facilities (e.g., community centers) was severely limited. St. Paul’s Hospital (SPH), a quaternary acute care hospital located in downtown Vancouver, is the primary hospital caring for individuals living in the DTES.

### 1.2. Outbreak Identification

Three individuals residing in DTES shelters presented to the emergency department (ED) with colitis on 20 February 2021. We identified *Shigella flexneri* in stool cultures from two patients, while the third patient was diagnosed with *Shigella flexneri* bacteremia. We notified Public Health and initiated an investigation and management plan for a potential outbreak.

### 1.3. Objectives

The aim of this study was to describe the clinical, epidemiologic, diagnostic, and genomic sequencing investigations of the *Shigella flexneri* outbreak in the DTES, as well as the impacts on the acute care hospital during the COVID-19 pandemic.

## 2. Methods

### 2.1. Overview

After a cluster of three *Shigella flexneri* 2a infections was identified on February 21, 2021, we searched the laboratory database for cases of *Shigella flexneri* 2a diagnosed in the preceding six months that exhibited the same antibiogram results as the isolates in the initial cluster. We reviewed cases diagnosed at our hospital laboratory and in community laboratories. In this observational cohort study, we followed the Outbreak Reports and Intervention Studies of Nosocomial Infection (ORION) reporting guidelines. An outbreak of shigellosis was officially declared by Public Health on 26 February 2021.

### 2.2. Case Findings and Data Collection

We reviewed the hospital medical records of laboratory-confirmed cases, collected demographic and clinical data, and attempted to interview patients to identify additional risk factors. Additional information collected included food sources, HIV and Hepatitis C serostatus, substance use disorder, psychiatric comorbidities, and men who have sex with men status.

Public Health outreach workers collected information on risk factors, identified contacts, and identified symptomatic people in shelters and SROs. Shigellosis is a reportable communicable disease based on the Public Health Act in British Columbia.

### 2.3. Case Definitions

*Confirmed case of Shigellosis*: Isolation of *Shigella flexneri* 2a by microbial culture or detection of *Shigella*/enteroinvasive *E. coli* (EIEC) in a stool specimen by using culture-independent diagnostic testing (CIDT).

*Suspect case of Shigellosis*: A clinically compatible case that is temporally and spatially linked to the outbreak based on homelessness or poorly housed situations (shelter/SRO) during the outbreak time period.

### 2.4. Laboratory Methods

Stool specimens were collected in a sterile container or with rectal swabs (FecalSwab^TM^ Copan, Murrita, CA, USA). Standard bacterial cultures were completed following standardized microbiology protocols, and identification was performed using Vitek2 ID (bioMérieux, Marcy l’Etoile, France) or a Biofire FilmArray GI Panel [Biofire Diagnostics, Salt Lake City, UT, USA] (in select cases when rapid identification was necessary), with confirmation of *Shigella flexneri* via Polyvalent Agglutination Sera (Remel, Lenexa, KS, USA). We performed antibiotic susceptibility testing for ampicillin, trimethoprim–sulfamethoxazole, ciprofloxacin, ceftriaxone, and azithromycin, adhering to the guidelines of the Clinical Laboratory Standards Institute [6]. *S. flexneri* serotyping was performed at the BCCDC Public Health Laboratory (Vancouver, BC, Canada).

*Genome sequencing*: All culture-positive *S. flexneri* underwent whole-genome sequencing. Isolates were grown in 3 ml Mueller–Hinton broth overnight, then extracted on MagNA Pure 24 (Roche Diagnostics, Rotkruez, Switzerland). The sequencing library was prepared according to the SQK-LSK109 sequencing kit with 1D native barcoding kits, EXP-NBD104/114, and run on GridION with R9.4.1 flow cells (Oxford Nanopore Technologies, Oxford, UK). Raw sequencing data were base-called with Guppy (v4.1.0) and uploaded to BugSeq for further analysis [7]. In brief, reads were preprocessed, removing reads with mean Phred scores below 8 and lengths less than 100 bp. Reads passing quality control were assembled and polished [8,9,10]. Assembly quality was assessed using the *Shigella flexneri* 2A genome (NCBI Assembly GCA_000006925.2) as a reference [11].

Multilocus sequence typing of assembled genomes was determined, and serotype was predicted with SerotypeFinder [12]. *ShigEiFinder* was used to determine *Shigella* clusters [13]. Core-genome MLST was performed using the default scheme that was included (Enterobase (v1) [14]. In each pairwise comparison of isolates, alleles not found with exact matches to the cgMLST scheme were masked [15]. Antimicrobial resistance was predicted by BugSeq.

### 2.5. Epidemiologic and Statistical Analyses

We summarized clinical characteristics using descriptive statistics. Continuous variables were analyzed as medians and interquartile ranges. Binary variables were reported as percentages. We constructed the epidemic curve using R version 4.1.1 and the ggplot2 package.

### 2.6. Countermeasures

#### 2.6.1. Hospital-Based Interventions

On 23 February 2021, we informed relevant hospital services about *Shigella flexneri* infections among individuals living in the DTES. We alerted them to consider shigellosis on the differential diagnosis for those presenting with dysentery symptoms. The laboratory facilitated specimen collection using rectal swabs if stool collection was challenging or not feasible. Additionally, we highlighted pre-emptive treatment using azithromycin or ceftriaxone for patients at elevated risk and those who had challenges with follow-up care. For admitted patients, we prioritized single-room accommodations, emphasized hand hygiene, and ensured enhanced cleaning protocols to prevent nosocomial transmission.

#### 2.6.2. Community-Based Interventions

We customized an outbreak investigation case report questionnaire to help track clinical information and potential exposures and contacts. Community outreach started on 26 February 2021 with alerts sent to shelters, outreach workers, and safety net providers in the community. We also posted an update on the local Public Health website to alert primary care and emergency room providers [16]. Environmental Health Officers inspected food service provider organizations. The Public Health team contacted homeless service providers (emergency shelters, day centers, supportive housing, food services, and encampments) whenever new cases were diagnosed. In addition to identifying suspected cases, they provided guidance on informing staff and facilities on sanitation and disinfection. In some settings (health clinics and tent encampments), azithromycin 500 mg for 3 days, or 1 g PO once, was given to any patient presenting with a syndrome compatible with shigellosis. We also provided instructions and supplies for diagnostic testing using self-collected rectal swabs in Cary–Blair media.

The city of Vancouver conducted street flushes in the alleys and streets of the Vancouver DTES in the month of May 2021. Portable toilets, hand hygiene stations, and potable water units were set up in May 2021.

### 2.7. Ethics

Ethics review was waived by the University of British Columbia/Providence Health Care research ethics board because the clinical work to investigate this outbreak is part of routine work conducted by IPAC in collaboration with Public Health.

## 3. Results

**Figure 1:**
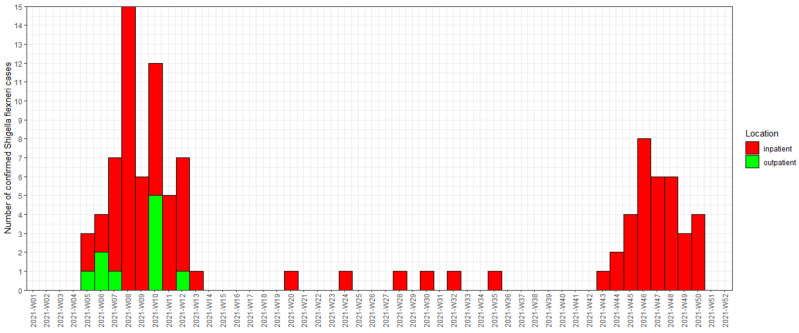
Epidemic curve showing episode date of *Shigella flexneri* 2a in the 2021 outbreak affecting PEH in the Vancouver DTES. We used the symptom onset when available for episode date. When this was not available (*n* = 5), the date of laboratory-confirmed specimen collection was used for the episode date. Inpatients included those who were admitted to the hospital or assessed in the emergency department. Outpatients included those who only had health care encounters in the community and were not seen in the emergency department or admitted to the hospital.

We identified a total of 101 confirmed cases of *Shigella flexneri* 2a from 31 January 2021 (the first laboratory-confirmed case) to 16 December 2021. Figure 1 shows the epidemic curve of the *Shigella flexneri* 2a outbreak affecting residents of the DTES. The outbreak was officially declared to be concluded on 19 May 2021, but a resurgence of cases was noted in October and November. The number of suspected cases could not be systematically ascertained because of the high number of community sites seeing patients and many prescribing empiric treatments without collecting stool samples or rectal swabs.

Of the 101 confirmed cases, 81 were male (80%). The median age was 43 years (IQR 33–55). None of the cases had stable housing. Ninety-one patients were either admitted to SPH or seen as outpatients in the ED. Many patients were brought in by emergency medical services after being found down on the streets, covered in stool and vomit. Further details on the hospital length of stay, symptoms, time from symptom onset to specimen collection, and comorbidities are shown in Table 1.

None of the patients had a documented travel history, and none of them reported that they were sexually active in the preceding month prior to the date of diagnosis. Although Environmental Health Officers visited and inspected the kitchens of volunteer organizations and reviewed to see if any staff had symptoms of shigellosis, many individuals obtained food from volunteer organizations in the DTES and from garbage bins, and no obvious food source could be identified.

Secondary *S. flexneri* bacteremia was identified in five cases. Three patients required admission to the intensive care unit (ICU). We identified one death within 30 days of diagnosis in a patient who was hospitalized and developed bacteremia. We did not follow up with each case to determine if they developed post-infectious complications (e.g., reactive arthritis).

For antimicrobial treatment, most patients received recommended antibiotics (azithromycin or ceftriaxone). For patients who initially received antibiotics in the hospital, we did not follow up to see if outpatient prescriptions were filled or taken. Also, we were unable to track how many courses of azithromycin were distributed by outreach teams to people in community clinics and high-risk settings such as the tent encampment site at one of the local parks.

### 3.1. Whole-Genome Sequencing (WGS), Serotyping, and MLST

We sequenced 64 isolates and assembled a median of 28 contigs per isolate. Forty-four isolates had their genome completely assembled into a circular 4.6 megabase contig. All 64 sequences were identified as *Shigella flexneri* by BugSeq, and these spanned a median of 98.0% (minimum: 90.5%) of the *S. flexneri* genome.

All *S. flexneri* genomes were identified as serotype 2a and sequence type (ST) 245 by in silico serotyping and MLST, respectively. ShigEiFinder identified all isolates as cluster three. With core-genome MLST (cgMLST) on our isolates, we identified a mean of 2462 (minimum: 2255) exact matches to reference cgMLST alleles per study genome. We performed 1225 pairwise cgMLST profile comparisons between all 64 isolates: the median cgMSLT distance was 0 alleles (25th percentile: 0, 75th percentile: 3). The maximum distance between any two isolates was 14 alleles; however, all isolates had less than 10 cgMLST alleles (the PulseNet threshold for epidemiological relatedness) compared to at least one other *S. flexneri* isolate in our study.

### 3.2. Genotypic Antimicrobial Resistance

We correlated genotypic antimicrobial resistance (AMR) prediction with phenotypic AMR. All *S. flexneri* isolates shared the same genotypic resistance prediction to first-line drugs trimethoprim–sulfamethoxazole (TMP-SMX), fluoroquinolones, and ampicillin. Additional resistance was predicted for all isolates to amikacin, tobramycin, chloramphenicol, and tetracycline. The AMR genes are detailed in Table 2.

## 4. Discussion

The MDR *Shigella flexneri* 2a outbreak in the Vancouver DTES highlighted that high-income countries can be susceptible to large outbreaks of shigellosis in the setting of destitution, inadequate access to potable water and sanitation, and crowded living conditions. During the COVID-19 pandemic, these inequities were amplified. People experiencing homelessness faced increasing barriers to shelters and basic hygiene facilities. For those who were living in shelters or SROs, the congregate living environment also predisposed individuals to increased risks of exposure. Food insecurity could have compounded this problem as some people relied on garbage bins for food, which may have been contaminated with *Shigella* during the outbreak, although this was never confirmed. Although an initial source could not be proven, chains of transmission suggest propagation from inadequate hygiene and sanitation infrastructure (showers, toilets, and sinks with soap and running water). In contrast to transmission dynamics of shigellosis in the MSM community through person-to-person spread from sex, this outbreak appears to have propagated through ongoing contamination of the environmental surfaces and possibly unsafe food [17]. There was heavy rain in both January and November 2021, and heavy precipitation has been identified as a risk factor in similar shigellosis outbreaks before [18]. This may have contributed to the two peaks of the outbreak.

In Canada, shigellosis is a nationally notifiable disease. In British Columbia, between 2014 and 2020, the peak annual incidence in BC was 3.9 cases per 100,000 population in 2019. In 2021, during the outbreak, the incidence was 4.3 per 100,000 population in BC, and 13.6 per 100,000 population in the Vancouver Coastal region. Since then, the incidence of shigellosis has continued to rise with *Shigella sonnei* [19,20].

Similarly, a prolonged outbreak of shigellosis among the inner-city population in another Canadian city (Edmonton, Alberta) was reported from September 2022 to February 2023. However, three weeks after the outbreak was declared over, cases increased, and the outbreak was ongoing up to October 2024, with 442 people confirmed to be infected and 69% of those individuals requiring hospitalization [21]. An outbreak of shigellosis was also reported among PEH in Seattle and King County at the end of October 2020 [22]. That outbreak lasted until May 2021 and had 117 confirmed cases of *S. flexneri*, of which 62% were hospitalized. Similar to our findings, a lack of access to clean drinking water, toilets, and handwashing facilities likely contributed to the spread of *Shigella*. Water sources and equipment in decorative fountains were tested within the Seattle downtown area, but the sources of the outbreak were not identified.

In terms of our outbreak response, some strengths included the rapid identification of the cluster and the dissemination of information to providers. We provided community clinics with rectal swabs for an easier collection of stool samples. The rapid identification of cases using the Biofire FilmArray GI panel also helped. We also communicated the importance of pre-emptive treatment in patients presenting with symptoms compatible with shigellosis. In a modeling study investigating the value of interventions in reducing total attack rate and shortening the duration of the outbreak, antibiotic treatment was the only single intervention strategy to have an effect [23]. Ciprofloxacin is typically recommended as a first-line treatment, but given the multidrug-resistant outbreak strain, only azithromycin was available as an oral therapy option. Challenges with drug therapy adherence and follow-up in our population necessitated a recommendation of a single 1 g dose of azithromycin as a first-line treatment [24,25]. Despite testing susceptibility, there is limited evidence supporting the use of cefixime and concerns for higher failure rates [26,27]. Hence, cefixime was recommended only when other options were not possible. In patients who were immunocompromised or hospitalized with severe disease, we recommended ceftriaxone [28].

We applied WGS tools and analyses to demonstrate the clonal spread in this outbreak. Historically, Oxford Nanopore sequencing has had a high sequencing error rate, precluding its use for detailed outbreak investigation [29]. Recently, advances in sequencing chemistry, base-callers, and assembly algorithms have led to dramatic increases in raw read and assembly accuracy [30]. To our knowledge, we are one of the first groups to perform outbreak analyses and cgMLST using only whole-genome nanopore sequencing. Xu et al. attempted to perform cgMLST on *Salmonella* isolates using nanopore sequencing; however, sequencing errors led them to rely on the more granular serotyping to infer relatedness instead of cgMLST [31]. Similarly, González-Escalona et al. had to polish their nanopore assemblies with Illumina reads to derive accurate cgMLST distances [32]. We attribute our success in performing cgMLST on ONT sequencing data to several factors. First, we used the latest ONT sequencing kits and base-caller at the time of the analyses (September 2021). Second, BugSeq’s assembly pipeline uses an evidence-based pipeline to maximize consensus sequence quality.

We were limited by our ability to interview all affected individuals in a timely manner to reduce recall bias. We largely relied on chart reviews where missing information could not be imputed. We were also unable to track the number of suspected epidemiologically linked cases because of the number of providers involved in assessing patients in the community and the number of people at risk who have transient interactions with the healthcare system. In one study using a susceptible–infected–recovered time series model, it was estimated that, at most, 20% of cases are reported [33]. Underreporting is affected by three main factors: the ill person needs to seek medical care, a specimen needs to be collected properly, and the testing laboratory needs to be equipped to perform sensitive diagnostics. In this outbreak, despite our highly sensitive diagnostic tools, the first part of the chain in seeking care is challenging for PEH. Another area for improvement in our response is to expeditiously deploy hygiene and sanitation infrastructure (toilets, hand hygiene stations, and showers). Although we notified shelters and SROs of confirmed cases in people housed there, resources to enhance cleaning of the facilities were limited.

## 5. Conclusions

In high-income countries, PEH and those who live in poor and unstable housing conditions face high risks for shigellosis. COVID-19 has exacerbated longstanding public health concerns around the dearth of hygiene and sanitation resources available to PEH. Preventing similar outbreaks will require mitigating these risk factors and finding solutions to the crisis of homelessness in Canada.

## Figures and Tables

**Table 1:** Summary of patient characteristics and demographics. The denominators for each variable are listed (*n* = 101 for the cohort; *n* = 90 for the hospital cohort).

Characteristics	Number
Median age (*n* = 101)	43 (IQR 33–55)
Sex (*n* = 101)	
Male	81 (80%)
Female	20 (20%)
Housing situation (*n* = 101)	
Living on the streets or in tent encampments	24 (23.7%)
Using shelters	24 (23.7%)
Single-room-occupancy hotel	53 (52.5%)
Place of diagnosis (*n* = 101)	
Emergency department	90 (89.1%)
Community outpatient clinic	11 (10.9%)
Median days of hospital stay (*n* = 90)	3 (IQR 1–5)
Number of patients with *Shigella flexneri* bacteremia (*n* = 90)	5 (5.6%)
Admitted to ICU (*n* = 90)	3 (3.3%)
Median days from symptom onset to specimen collection (*n* = 90)	3
Symptoms (*n* = 90)	
Diarrhea	82 (91.1%)
Abdominal pain/cramps	62 (68.9%)
Nausea	45 (50%)
Vomiting	41 (45.6%)
Bloody stools	35 (38.9%)
Medical imaging (*n* = 90)	
Evidence of colitis (mild to severe)	18 (20%)
No evidence of colitis	48 (53.3%)
Not performed	24 (26.7%)
Comorbidities (*n* = 90)	
HIV-seropositive	19 (21.1%)
Median CD4 count (cells/mL) for cases with HIV	235
Hepatitis C-seropositive	37 (41.1%)
Substance use disorders (fentanyl, heroin, crack cocaine, and methamphetamines)	80 (88.8%)
Psychiatric comorbidities	13 (14.4%)
Men who have sex with men (MSM)	1 (1.2%)
Number of patients who received antimicrobial treatment (*n* = 90)	85 (94.4%)

**Table 2:** Antimicrobial resistance genes identified for *S. flexneri* isolates.

Antimicrobial or Antimicrobial Class	Resistance Genes
TMP-SMX	*dfrA1* and *sul2* genes
Ampicillin	*bla*OXA-1 and *bla*TEM-30
Fluoroquinolones	*gyrA* mutation (S83L) and aac(6′)-Ib-cr
Amikacin/Tobramycin	aac(6′)-Ib-cr
Chloramphenicol	*CatB3* and *catA1*
Tetracycline	*tetA* and *tetB*

## Data Availability

The original contributions presented in this study are included in the article. Further inquiries can be directed to the corresponding author.
